# Genetic Disruption of Both Tryptophan Hydroxylase Genes Dramatically Reduces Serotonin and Affects Behavior in Models Sensitive to Antidepressants

**DOI:** 10.1371/journal.pone.0003301

**Published:** 2008-10-15

**Authors:** Katerina V. Savelieva, Shulei Zhao, Vladimir M. Pogorelov, Indrani Rajan, Qi Yang, Emily Cullinan, Thomas H. Lanthorn

**Affiliations:** Lexicon Pharmaceuticals Incorporated, The Woodlands, Texas, United States of America; University of Parma, Italy

## Abstract

The neurotransmitter serotonin (5-HT) plays an important role in both the peripheral and central nervous systems. The biosynthesis of serotonin is regulated by two rate-limiting enzymes, tryptophan hydroxylase-1 and -2 (TPH1 and TPH2). We used a gene-targeting approach to generate mice with selective and complete elimination of the two known TPH isoforms. This resulted in dramatically reduced central 5-HT levels in *Tph2* knockout (TPH2KO) and *Tph1/Tph2* double knockout (DKO) mice; and substantially reduced peripheral 5-HT levels in DKO, but not TPH2KO mice. Therefore, differential expression of the two isoforms of TPH was reflected in corresponding depletion of 5-HT content in the brain and periphery. Surprisingly, despite the prominent and evolutionarily ancient role that 5-HT plays in both vertebrate and invertebrate physiology, none of these mutations resulted in an overt phenotype. TPH2KO and DKO mice were viable and normal in appearance. Behavioral alterations in assays with predictive validity for antidepressants were among the very few phenotypes uncovered. These behavioral changes were subtle in the TPH2KO mice; they were enhanced in the DKO mice. Herein, we confirm findings from prior descriptions of TPH1 knockout mice and present the first reported phenotypic evaluations of *Tph2* and *Tph1/Tph2* knockout mice. The behavioral effects observed in the TPH2 KO and DKO mice strongly confirm the role of 5-HT and its synthetic enzymes in the etiology and treatment of affective disorders.

## Introduction

The neurotransmitter serotonin (5-HT) has been implicated in a variety of physiological functions in both the peripheral and central nervous systems. In mammals, serotonergic neurotransmission regulates a wide variety of neurobehavioral processes, including cognition, affective states, ingestive behavior, motor control, and sensorimotor integration [1]–[6]. Serotonin is also an important modulator of the gastrointestinal system via the enteric nervous system [7]. Given the wide range of functions that it modulates, one might expect the depletion of 5-HT to have serious consequences for development and maintenance of viability: in fact some evidence exists that peripheral serotonin is important for proper embryonic development [8].

Impaired or altered 5-HT neurotransmission appears to be a central dysfunction leading to depressive and anxiety symptoms. Mutations in TPH2 have been associated with a number of such disorders and the data is controversial [9]–[14]. Moreover, a number of primary therapeutics for depression, anxiety, and some neurological conditions, such as emesis and irritable bowel syndrome, affect the activity of the 5-HT system [15]–[17].

5-HT is synthesized in two steps from the amino acid tryptophan with tryptophan hydroxylase (TPH) being the rate-limiting enzyme. Until recently it was thought that only one isoform of TPH existed. However, genetic deletion of the classical enzyme did not appreciably reduce 5-HT content in the brain, spurring a successful search for an additional enzyme [18]. It is now established that two isoforms exist. Tryptophan hydroxylase-1 (TPH1), the long-recognized isoform, is responsible for the synthesis of most peripheral 5-HT, whereas tryptophan hydroxylase-2 (TPH2) is neuronal specific [19], [20] and therefore likely to control most central 5-HT synthesis.

We used a gene-targeting approach to generate mouse lines lacking either *Tph1* or *Tph2*, and then crossbred the two lines to produce animals lacking both genes. Here we report findings from *Tph1* and *Tph2* single knockout (KO) mice, and *Tph1/Tph2* double knockout (DKO) mice. To our knowledge this is the first report with full phenotypic evaluation of mice with genetic deletion of *Tph2* and of both *Tph* isoforms.

## Materials and Methods

### Generation of TPH1, TPH2 and TPH1/TPH2 deficient animals

We constructed the *Tph1* (NM_009414) and *Tph2* (NM_173391) targeting vectors from the lambda KOS™ system [21]. The yeast selection cassette was generated by PCR to include mouse genomic sequences flanking the region of deletion and introduced into the respective mouse genomic clone by yeast recombination. Homologous recombination resulted in the deletion of coding exon 2 of *Tph1* and coding exons 1 and 2 of *Tph2*. The *Not*1-linearized targeting vector was electroporated into 129S5/SvEvBrd embryonic stem (ES) cells. G418/FIAU resistant ES targeted clones were isolated and confirmed by southern hybridization on both arms of homology with gene specific probes. In addition a Neo probe was used to screen for random insertion events. Targeted ES clones of each deletion were independently injected into C57BL/6J-*Tyr* ^c-Brd^ (albino) blastocysts and the resulting chimeras were mated to C57BL/6J-*Tyr* ^c-Brd^ females to generate F1 heterozygous mice for each KO line. For single gene-deletion phenotypic analyses, the F1 heterozygous progeny are intercrossed to generate F2 wild type (WT), heterozygous (Het), and homozygous (KO) progeny for each of the two *Tph* genes. WT, Het, and KO progeny were obtained in normal Mendelian ratios (1∶2∶1) for each of the *Tph* genes.

To generate *Tph1/Tph2* DKO mice, *Tph1* KO males were bred with *Tph2* Het females. Animals heterozygous for both loci were selected from the progeny, and subsequent breeding of double heterozygotes was undertaken to produce DKO animals, which were recovered at Mendelian ratios (one in sixteen).

All experiments described below were carried out with protocols approved by The Institutional Animal Care and Use Committee of Lexicon Pharmaceuticals.

### Genotyping

Genotyping of tail DNA by PCR was conducted according to standard protocols [22]. TPH1KO mice were genotyped with the following primers: P1: 5′ACC ACC TTC TTC CTC CTT TTG AGC 3′ and P2: 5′ CCG GGA GAT CTG TGA AGA GTT TGG 3′ that generated a 654 bp PCR product in the WT (+/+) and Het. Mutant specific strategy produced a 483 bp amplicon in the Het and Hom with the following primer pair: P3: 5′ GCA GCG CAT CGC CTT CTA TC 3′ and P4: 5′ CCG GGA GAT CTG TGA AGA GTT TGG 3′.

To genotype TPH2KO mice we used the following primer pairs: for wild type band (+/+) P5: 5′TTA TTA CCC ACA AAC GCT GAA 3′ and P6:5′GTT TAT GCT GGC ACT GGT ACT TGA 3′ that generated a 348 bp amplicon; for the mutant band P7: 5′GCA GCG CAT CGC CTT CTA TC 3′ and P8: 5′CCT TAA TGA CAG GGA ACA AGT C 3′ generated a 235 bp PCR product.

DKO mice were genotyped with the same primer sets as was used for each individual gene KO in a multiplex reaction.

### Subjects

Animals used for all studies were male and female KO or DKO and WT cohort mates bred in a mixed (C57BL/6J-*Tyr* ^c-Brd^×129S5/SvEvBrd) genetic background at Lexicon Pharmaceuticals. All mice were maintained under a standard light/dark cycle from 7 am to 7 pm. They were housed in groups of five in 30×20×20 cm acrylic cages with food and water freely available. For TPH1KO the cohort consisted of 12 female KO, 18 male KO, 10 female WT and 6 male WT. This cohort was run through a standard phenotypic analysis battery used at Lexicon Pharmaceuticals (as an example, the phenotypic screen of VGLUT1 mice is accessible at: http://www.informatics.jax.org/external/ko/lexicon/2383.html). For TPH2KO there were three separate cohorts of mice used in the experiments. Cohort 1 was run through a comprehensive phenotypic analysis and consisted of 4 male and 4 female mice per genotype. Cohort 2 consisted of 11 female KO, 6 male KO, 11 female WT and 9 male WT. Cohort 2 was tested in a standard behavioral phenotypic analysis battery at age of 12 weeks. Cohort 3 consisted of 15 female KO, 17 male KO, 19 female WT and 15 male WT. Cohort 3 was tested in the marble burying, platform anxiety test, and forced swim test at 12–14 weeks of age, and males from Cohort 3 were also tested in the repeated tail suspension test when mice were 6 months old. Male mice from cohort 3 were then used for detection of neurotransmitter levels in different brain regions. For TPH1/TPH2 DKO mice the cohort consisted of 12 female DKO, 6 male DKO, 9 female WT and 10 male WT and was tested in a standard behavioral phenotypic analysis battery starting at age of 12 weeks.

### Neurochemical Analysis

To analyze the contents of monoamine neurotransmitters in various regions of the brain mice were sacrificed for tissue dissection. The olfactory bulb, cerebellum, brainstem, hippocampus, striatum, cortex, and thalamus with hypothalamus were dissected out and frozen immediately on dry ice. The brain tissues were subsequently homogenized in 4× volume (i.e. 4 µL of buffer for each mg of tissue) of ice-cold buffer (0.1 M trichloroacetic acid, 10 mM sodium acetate, 0.1 mM EDTA, pH 3.7) in glass homogenizers. The homogenates were transferred to an Eppendorf tube and centrifuged at 18000× g at 4°C for 5 min. The supernatants were collected and passed through a 0.22 µm filter column and then through a filter column with a molecular weight cutoff at 10K (Nanosep 10K; Pall Life Sciences, East Hill, NY) by centrifugation at 18000× g at 4°C. To analyze the contents of dopamine, norepinephrine, serotonin and their metabolites in the tissue extracts, 15 µL of the extract from each tissue was injected into an HPLC system (Waters 515 pump, Water Corporation, Milford, MA) with an C18 column (MD-150 C18 column, ESA Inc., Chelmsford, MA) and an electrochemical detector (Coulochem III, ESA Inc., Chelmsford, MA).

### Peripheral 5-HT levels

Mice were anesthetized using isoflurane during blood draw via cardiac puncture and then sacrificed by rapid decapitation. Mid-jejunum and entire colon were isolated, mesenteric fat removed, gut lumen opened and blotted dry, and tissues frozen immediately in liquid nitrogen and stored at −80 C for further analysis. 5-HT was extracted from the tissues by homogenizing in a buffer containing 300 mM trichloroacetic acid, 100 mM sodium acetate, pH 3.5, 10 mM EDTA, and 20 mM sodium bisulfate. The homogenates were centrifuged and supernatants analyzed for 5-HT content using an HPLC method.

### Histology

Tissues were collected from TPH1KO (n = 8; 18–20 weeks of age), TPH2KO (n = 2; 16 weeks of age) and DKO mice (*n* = 2; 49 to 51 weeks of age) as well as age-matched WT mice and were fixed by immersion in 10% neutral buffered formalin. All tissues were embedded in paraffin, sectioned at 4 µm, and mounted on positively charged glass slides (Superfrost Plus, Fisher Scientific, Pittsburgh, PA) and stained with hematoxylin and eosin for histopathologic examination. For the full list of tissues examined see Table S1.

### DEXA analysis

Mice were anesthetized by intraperitoneal injection of Avertin (1.25% 2,2,2,-tribromoethanol, 20 ml/kg body weight ), measured body length and weight, and then placed in a prone position on the platform of the PIXImus™ Densitometer (Lunar Inc.) for a DEXA scan. Using a Lunar PIXImus software, the Bone mineral density (BMD) and fat composition (% fat) and total tissue mass (TTM) were determined in the regions of interest (whole body, vertebrae, and both femurs).

### Behavior assays

Mice were tested in a standardized behavioral phenotyping battery. A comprehensive phenotypic analysis (including a subset of behavioral tests derived from the Irwin screen) revealed no notable abnormalities across a wide range of behaviors as well as assays for cardiac, immune system, endocrine, and ophthalmic function (for the full list of assays see Table S2). Methods not described in the main text are available in Methods S1.

#### Locomotor activity

Locomotor and exploratory behaviors were recorded with twelve Digiscan open field (OF) apparatus and Versamax software, v.4.00-127E (Accuscan Instruments, Inc., Columbus, OH). A large arena (42 cm×42 cm) with infrared beams at three different levels was used to record horizontal locomotor activity (total distance), vertical movement (rearing), and investigation into the 4 holes in the floor of the open-field (hole poke). Two florescent lamps positioned over each chamber provided light levels of 800 lux in the center of each open field arena. Each animal was placed in the center of the open field and its activity was measured for 20 min. The total distance traveled (cm), vertical movement number (rearing), number of hole pokes, time spent in the center of the OF (time-in-center), distance traveled in the center of the OF (center distance), and center/total distance ratio over the intervals were recorded using Versadat program, v.2.70-127E (Accuscan Instruments). The OF center area measured 20×20 cm.

#### Marble burying test

The marble burying test for anxiety-related and compulsive-like behaviors was performed as previously described [23]. Mice were individually placed into cages (24×18×13 cm) filled to a depth of 5 cm with clean bedding for a 30-min testing period. Prior to each testing round, the experimenter evenly spaced 25 identical marbles across the bedding surface. 16 mice were examined in separate cages during each testing round. After 30 min, each mouse was returned to its home cage and all marbles 2/3 or more covered by bedding were scored as buried by an experimenter blind to genotype.

**Hot Plate test** to assess nociceptive response to an acute thermal pain stimulus was performed using a Columbus Instruments Hotplate Analgesia meter model 1440 (Columbus, OH). The hot plate was set to 55° C and controlled to within 0.1° C. The size of the heated surface area was 10″×10″×0.75″. The response time for each animal (hind-paw flinching) was recorded by the experimenter (blind to test subject genotype).

#### Tail Suspension test

The tail suspension test for depression-related behavior was conducted using 8 chambers from Med Associates Inc (PHM-300TSS Mouse Tail Suspension System, Med Associates, Georgia, VT). The mouse was securely fastened with medical adhesive tape by the tip (∼1.0–1.5 cm) of the tail to a metal hanger and suspended above the floor in a visually isolated cubicle. The data was recorded using Med Associates Tail Suspension software version 2 (Med Associates, Georgia, VT). Immobility was defined as the area under the curve using an immobility threshold of 2. The total duration of immobility over a single 6-min session was recorded for each mouse. For *Tph2* KO male mice the test was repeated for three days. Animals that climbed their tails during testing on any of the test days were excluded from data analysis.

#### Forced swim test

The FST chamber was a 2000 ml Pyrex beaker with a diameter of 14 cm filled with deionized water (25°C) to a depth of 14 cm. A black Plexiglas chamber (30 cm×28 cm×20 cm) surrounded each beaker. The front and top of the chambers were open to allow for video recording. To optimize contrast, a black background was used for white mice, and a white background was used for agouti and black mice. With respect to the forced swim test, our in-house experience showed that measuring immobility for one six-minute session (standard mouse one-day protocol) is not sufficient to detect an antidepressant-like effect for certain antidepressants. We therefore employed a 2-day protocol similar to that commonly used for rats [24], [25] and found this to enhance the sensitivity of the test [26].

On the first testing day mice swam for 15 min (pre-test session). Twenty four hours later, mice were exposed to the same experimental conditions for 5 min (test session). The water in the beaker was changed between each animal. The data was analyzed with image analysis software, VideoTrack Quantization (ViewPoint Life Sciences, Montreal, QC, Canada) and immobility and struggling were quantified for the duration of the first 5 min during pre-test (so that we also have data comparable to the standard one-day mouse FST) and all 5 min during the test session. Immobility was defined as minimal movements required for a mouse to keep its head above the water. Struggling was defined as vigorous movements with forepaws breaking the water. The same thresholds were set for all animals (<450 was considered immobility; >785 was counted as struggle). Swimming was defined in the program as activity measuring between thresholds 450 and 785, but was not used in analyses as it represented the difference between total test time and measures for immobility, and struggling.

### Data analysis

Results are presented as individual data points or as means +/− S.E.M. The Statistica 7.0 software package (StatSoft, Inc., Tulsa, OK) was used to determine significant differences between groups. The data was analyzed with one-way ANOVA tests with genotype and sex (where both sexes were tested) as main effects. Tukey's HSD or Dunnett's post hoc analyses were used when main effects or interactions were significant. Unpaired two-tailed t-tests were used to compare genotypes when data from males and females was analyzed separately, and where animals of only one sex were tested. The data for OF and repeated tail suspension tests was analyzed using repeated measures ANOVA with genotype and sex as main effects, and test interval or day as a repeated measure.

## Results

**Figure 1:**
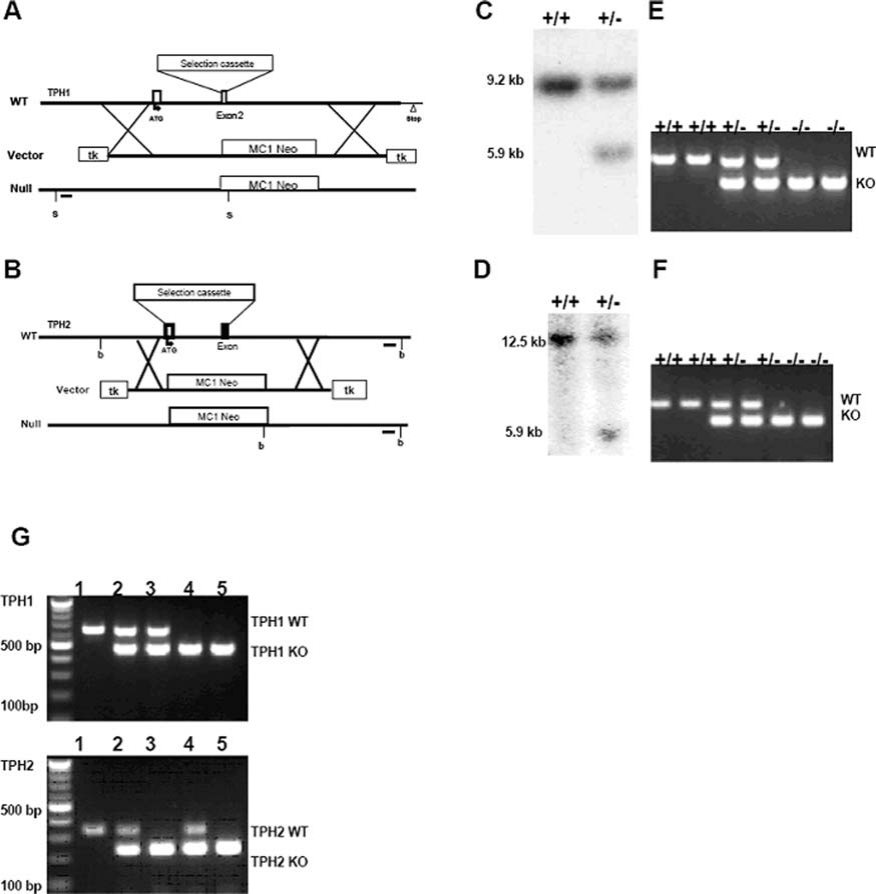
Generation and confirmation of *TPH1*, *TPH2* and *TPH1/TPH1* deficient animals. (A) Genomic organization of exon 1 and exon 2 of TPH1 gene (WT), the targeting construct (Vector), and the mutant allele (Null). The MC1 Neo selection cassette replaces exon 2 by homologous recombination. The recombined allele is shown as the null allele with the restriction enzyme of choice Spe1 (s) and the 5′ external probe (black bar) used for southern hybridization. (B) Genomic organization of exon 1 and exon 2 of TPH2 gene, the targeting vector construct, and the mutant allele. The MC1 Neo selection cassette replaces exons 1 and 2 by homologous recombination. The recombined allele is shown as the null allele with the restriction enzyme of choice Bgl1 (b) and the external probe (black bar) used for southern hybridization. (C) TPH1 targeted ESC genomic DNA was restriction digested with Spe1 and hybridized with the 5′ external probe to show the wild type (+/+) 9.2 kb band and the mutant band (+/−) at 5.9 kb in the heterozygous ES cells. (D) Targeted ES cell genomic DNA was restriction digested with Bgl1 (b) and hybridized with the 3′ external probe to show the wild type (+/+) 12.5 kb band and the mutant band (+/−) at 5.9 kb in the heterozygous ES cells. (E) The pups were genotyped by isolating tail genomic DNA and PCR amplifying sequences using specific TPH1 primers that amplified a 654 bp product in the wildtypes (+/+) and hets (+/−) and primers specific to the deletion insertion that produced a 483 bp product in the Hets (+/−) and the Homs (−/−). (F) The TPH2 null pups were genotyped by isolating tail genomic DNA and PCR amplifying sequences using specific primers that amplified a 348 bp product in the wildtypes (+/+) and hets (+/−) and primers specific to the deletion insertion that produced a 235 bp product in the Hets (+/−) and the Homs (−/−). (G) The DKO pups were genotyped with the TPH1 and TPH2 specific primers as in Fig.1E, F. Individual animals (1–5), of each relevant genotype is shown: 1) TPH1^+/+^/TPH2^+/+^; 2) TPH1^+/−^/TPH2^+/−^, 3) TPH1^+/−^/TPH2^−/−^; 4) TPH1^−/−^/TPH2^+/−^, 5) TPH1^−/−^/TPH2^−/−^. 100 bp molecular weight markers are also indicated.

A homologous recombination deletion strategy was utilized to replace coding exon 2 of *Tph1* (Fig. 1A) and coding exons 1 and 2 (Fig. 1B) of *Tph2* with a LacZ/neomycin resistance gene cassette. G418/FIAU resistant ESC clones for *Tph1* deletion were analyzed by southern hybridization of SpeI (s) digested genomic DNA blot with a 244 bp 5′ external probe (Fig. 1A) generated using: 5′GCT CTT CTA AAA CGT CCA GTA G 3′ and 5′GTC TGA GTA AGA TTA AAC AAT CCG 3′ as primer pairs. This 5′ external probe labeled a 9.238 kb band in SpeI digested WT genomic DNA and a 5.965 kb mutant band in the recombined genomic DNA (Fig. 1C). G418/FIAU resistant ES-cell clones for TPH2 deletion were analyzed by southern hybridization with a 217 bp 3′external probe (Fig. 1B) generated using the following primers; forward 5′ACA TAC CAA TTC AAC CCA ATG 3′ and reverse 5′AGA GGG AAG ATG CCA CAT AGA T 3′. This external probe labeled a 12.526 kb band in Bgl1 (b) digested WT genomic DNA and a 5.888 kb band in the deletion mutant (Fig. 1D). The germline male chimeric mice were crossed with C57BL/6J female mice to generate the single gene homozygous, heterozygous and WT offspring as confirmed by PCR analysis (Fig. 1 E, F). Subsequent breeding was undertaken to produce double heterozygotes which were bred to generate double knockouts deleting both *Tph1* and *Tph2* genes as confirmed by genotyping analysis (Fig. 1G).

TPH2KO and DKO mice were analyzed by HPLC for levels of monoamine neurotransmitters and their metabolites in various regions of the brain. Levels of 5-HT and its metabolite, 5-HIAA, were dramatically reduced in all brain regions examined both in TPH2KO and DKO animals (Table 1). Reduction of 5-HT in TPH2KO mice ranged from 67.5% (cerebellum) to 96.9% (striatum), while 5-HT reduction in DKO mice ranged from 94.4% (cerebellum) to 99.2% (cortex). 5-HT levels were lower in DKO mice than in TPH2KO mice in all brain regions examined. The percentage of 5-HIAA reduction paralleled changes in 5-HT. No generalized changes were noted in other neurotransmitter levels (Table 1).

**Table 1:** Monoamine neurotransmitters and their metabolite levels (mean±S.E.M., pmol/g tissue) in different brain regions.

Brain Regions		Ctx	Th	Ob	Cb	Hipp	Bs	St
5-HT	WT	756±94	1100±71.2	293±42.1	103±16.8	506±54.3	702±96	517±64.6
	TPH2KO	62.4±7.1* (93.1%)	36.2±2.4* (96.7%)	22.6±1.94* (92.7%)	37.7±7.96* (67.5%)	19.4±3.46* (96.2%)	97.6±20* (94.8%)	15.8±1.25* (96.9%)
	DKO	6.52±2*^†^ (99.2%)	18.6±2.8*^†^ (98.3%)	4.98±2.50*^†^ (98.4%)	6.45±3.95*^†^ (94.4%)	8.01±3.5* (98.4%)	10.3±2.2* (98.6%)	8.65±1.26* (98.3%)
5-HIAA	WT	507±75.7	1398±234	373±78	205±31.5	724±126	818±119	535±102
	TPH2KO	21.4±3.4* (95.8%)	19.1±2.4* (98.6%)	29.5±5.9* (92.1%)	29.9±6.31* (85.4%)	13.1±1.61* (98.2%)	24.7±2.1* (98.1%)	9.4±1.2* (99.0%)
	DKO	9.35±3.35* (98.1%)	9.27±3.7* (99.3%)	4.95±1.75* (98.7%)	2.22±1.02* (98.9%)	4.87±1.58* (99.3%)	4.3±1.5* (99.5%)	3.6±1.04* (99.3%)
NE	WT	744±64	1761±142	1512±189	907±117	1530±89	1512±99	661±113
	TPH2KO	733±76	1352±115	1483±195	921±123	1459±157	1542±203	938±148
	DKO	618±58	1540±300	831±86	543±83	1540±268	1248±106	215±27^†^
NM	WT	169±43	119±15	261±26	211±22	133±15	57±11	210±145
	TPH2KO	69±16	78±14	279±18	205±24	166±22	87±18	n/a
	DKO	224±37	153±23	406±0 #	296±58	218±25*	55±21	231±162
DA	WT	2292±351	838±118	607±77	121±34	211±104	219±38	24961±2616
	TPH2KO	2529±271	492±37	507±90	93±40	67±21	134±15	24890±1115
	DKO	1640±135	2168±689*^††^	789±135	179±69	108±15	181±46	n/a
DOPAC	WT	1442±134	951±107	1076±213	230±38	244±48	376±52	6341±848
	TPH2KO	1683±142	998±120	1399±279	211±31	267±47	525±97	8196±833
	DKO	1062±160	1020±121	379±98	161±36	159±27	238±43	3886±546 ^†^
HVA	WT	650±74	532±79	387±70	60±6	94±11	164±19	2584±340
	TPH2KO	591±93	365±47	289±55	57±8	87±18	135±10	2194±350
	DKO	958±180	985±52*^††^	662±133^†^	68±11	145±13	212±17^†^	3864±355 ^†^

5-HT = 5-hydroxytryptophan (serotonin); 5-HIAA = 5-hydroxyindoleacetic acid; NE = norepinephrine; NM = normetanephrine; DA = dopamine; DOPAC = 3,4-dihydroxyphenylacetic acid; HVA = homovanillic acid. Brain regions examined: cortex (Ctx), thalamus/hypothalamus (Th), olfactory bulb (Ob), cerebellum (Cb), hippocampus (Hipp), brainstem (Bs), striatum (St). Figures in parentheses are percentages of reduction compared to WT controls. The numbers of animals used for the assays n = 16 for WT, n = 12 for TPH2KO, and n = 5 for DKO (all mice were males). 5-HT and 5-HIAA levels in all tissues from TPH2KO and DKO were significantly lower than those in WT (all p values<0.001 using one-way ANOVA with Tukey's post-hoc test). * - p<0.05 compared to WT (Tukey's post-hoc test).

^†^ - p<0.05, ^††^ -p<0.01 TPH1/TPH2 DKO compared to TPH2KO (Tukey's post-hoc test).

# - only one sample available due to signal loss (background noise).

Mice were also analyzed for peripheral 5-HT (Table 2). In TPH1KO mice 5-HT levels in blood, jejunum, and colon were strongly reduced, as expected [27]. TPH2KO mice did not differ significantly from WT mice in peripheral 5-HT levels. In DKO mice 5-HT was greatly reduced in these peripheral tissues.

**Table 2:** Comparison of peripheral 5-HT levels in TPH1KO, TPH2KO and TPH1/TPH2 DKO mice (mean±S.E.M.).

	Blood	Jejunum	Colon
	µM	fmol/mg	fmol/mg
WT n = 17	24.3±0.5	24763±514	107137±1357
TPH1KO n = 4	1.6±0.1*	378±30*	671±96*
TPH2KO n = 4	18.1±1.3	29000±2700	107025±2946 †
DKO n = 3	1.7±0.1*	73±6.2*	NT

* 5-HT levels are significantly lower than those in WT (p values≤0.01, one way ANOVA followed by Dunnett's post-hoc test).

† n = 10.

NT: Not Tested.

These changes in 5-HT and 5-HIAA levels demonstrate a marked disruption of brain 5-HT homeostasis in TPH2KO and DKO mice. To understand the role such changes may play in the pathophysiology of affective disorders, the mutant and WT mice from TPH1, TPH2, and DKO lines were tested in a series of behavioral assays selected to model different aspects of neurological disorders. TPH2KO and DKO mice did not differ from their WT cohort-mates in gross appearance, nor by histological analysis. Body size, weight, and percent body fat content were examined as a part of initial phenotypic evaluation (Table 3). Since an effect of gender on body length and weight was significant [F(1,62)>30, Ps<0.00001], males and females were analyzed separately. In males genotype effects were significant [F(3,27) = 4.4, 8.5, and 5.2 for body length, weight, and fat content, accordingly, p-values<0.05]. Post-hoc analysis revealed that body weight and length were significantly decreased in TPH2KO and DKO males, compared to WT males. In addition, DKO males also had decreased percent of body fat as measured by DEXA. In females a genotype effect on fat composition reached significance [F(3,35) = 6.0, p<0.01] with DKO females having significantly less body fat than WT controls.

**Table 3:** Body size, weight, and percent body fat (mean±S.D.) in WT, TPH1KO, TPH2KO, and TPH1/TPH2 DKO mice.

Sex/Genotype		Body Weight (g)	Body Size (cm)	% Body Fat
Males	WT (n = 18)	32.85±2.6	10.0±0.30	18.2±3.5
	TPH1KO (n = 4)	29.7±1.3	9.90±0.11	15.7±2.3
	TPH2KO (n = 4)	27.4±1.3 *	9.60±0.21 *	14.2±1.1
	DKO (n = 5)	29.2±1.7 *	9.68±0.20 *	13.0±0.2 **
Females	WT (n = 21)	25.18±3.9	9.46±0.30	21.0±5.5
	TPH1KO (n = 2)	23.39±1.6	9.3±0.14	15.8±1.9
	TPH2KO (n = 4)	22.4±2.9	9.12±0.40	15.3±0.5
	DKO (n = 12)	24.4±2.3	9.32±0.19	14.9±1.9 **

* p<0.05; ** p<0.01 compared with WT (Dunnett's post-hoc test).

There was no behavioral phenotype in TPH1KO mice in any of the assays, consistent with previously published data [19], [20], [28]. Therefore the behavioral data for TPH1KO mice are not discussed further.

For the TPH2KO and DKO, there were no differences between the KO or DKO and WT littermate control mice in motor coordination, acoustic startle response and sensorimotor gating, tonic inflammatory pain sensitivity, and learning and memory as assessed in inverted screen, pre-pulse inhibition, formalin paw, and trace fear conditioning assays, respectively (Table 4).

**Table 4:** General behavioral characterization of TPH1KO, TPH2KO and TPH1/TPH2 DKO mice.

	TPH1		TPH2		TPH1/TPH2	
	WT	KO	WT	KO	WT	KO
**Circadian (only females)**						
First 1 hour habituation	631±296 (10)	760±241 (12)	792±100 (2)	508±291 (4)	957±189 (9)	822±350 (12)
First 10 hours habituation	2737±1525 (10)	2785±973 (12)	2381±742 (2)	2374±764 (4)	3785±1385 (9)	4086±3333 (12)
Average light cycle activity	838±587 (10)	877±292 (12)	879±319 (2)	640±274 (4)	962±421 (9)	921±807 (12)
Average dark cycle activity	3514±2229 (10)	3185±488 (12)	3273±2070 (2)	3679±1993 (4)	4367±1949 (9)	4099±2869 (12)
**Open Field**						
Total distance (cm)	1861±648 (16)	2184±739 (30)	2228±839 (19)	1978±1070 (17)	2163±884 (19)	2553±875 (18)
Center Time Male	339±102 (6)	223±137 (18)#	490±143 (9)	344±192 (6)	337±138 (10)	322±88 (6)
Center Time Female	233±186 (10)	245±95 (12)	291±158 (10)	234±161 (11)	220±132 (9)	226±143 (12)
Rearing	43.3±36.4 (16)	57.6±38.3 (30)	54±32 (19)	61±40 (17)	69.1±53.2 (19)	78.3±31.6 (18)
**Basal Body Temperature**						
Female	35.6±0.53 (10)	35.8±0.75 (12)	36.1±0.62 (4)	36.9±1.0 (4)	36.4±0.37 (9)	36.8±0.45 (12)*
Male	35.0±0.74 (6)	35.8±0.6 (18)*	36.6±.56 (4)	36.7±0.61 (4)	36.4±0.78 (10)	36.5±0.94 (6)
**Stress-induced Hyperthermia**						
Only males	1.78±0.66 (6)	1.7±0.73 (18)	0.83±1.23 (4)	1.33±0.39 (4)	1.95±0.61 (10)	1.82±0.94 (6)
**Inverted Screen**						
Fell down (ratio)	0/16 (0%)	1/30 (4%)	1/8 (13%)	5/8 (63%)	1/19 (6%)	1/18 (6%)
Climbed up (ratio)	7/16 (44%)	20/30 (67%)	0/8 (0%)	6/8 (75%)	7/19 (37%)	11/18 (62%)
**Hot Plate**						
Latency to respond, sec	7.3±3.2 (16)	6.7±2.7 (30)	9.1±2.0 (8)	10.1±3.3 (8)	6.2±2.9 (19)	4.0±1.9 (18)*
**Acoustic Startle Response**						
120 dB	701±447 (16)	699±429 (30)	300±259 (8)	354±283 (8)	370±246 (19)	389±252 (18)
**PPI (%)**						
pp4	26.6±20.5 (15)	33±19.2 (27)	29.8±13.2 (7)	33.4±16.8 (6)	28.3±21.9 (14)	18.8±30.5 (17)
pp8	31.4±21.7 (15)	43.3±19.2 (27)	36.7±26.4 (7)	46.3±16.7 (6)	29.0±27.3 (14)	27.5±26.2 (17)
pp12	48.8±19.5 (15)	54.3±20.3 (27)	47.8±21.9 (7)	44.6±19.3 (6)	41.9±22.6 (14)	38.4±24.8 (17)
pp20	57.8±20.7 (15)	65.9±15.2 (27)	72.7±16.4 (7)	71.8±11.9 (6)	62.8±21.2 (14)	63.3±18.7 (17)
**Fear conditioning**						
Context freezing (%)	12.45±20.6 (16)	9.5±13.9 (30)	n/a	n/a	8.6±10.9 (19)	3.6±7.8 (18)
Cued freezing (%)	19.4±25.1 (16)	18.7±21.9 (30)	26.6±26.4 (8)	16.9±21 (8)	17.6±20.8 (19)	8.6±18.7 (18)
**Formalin Paw (only males)**						
Phase 1 flinches	251±54 (6)	173±66 (16)*	253±102 (4)	165±129 (4)	213±83 (10)	128±99 (6) #
Phase 2 flinches	624±208 (6)	452±187 (16)#	558±268 (4)	325±188 (4)	517±166 (10)	357±269 (6)

Data are expressed as mean±S.D. (N). Statistical analysis was performed for all assays. Mice with startle response below 100 were excluded from PPI analysis. * - p<0.05 from WT cohort mates (unpaired t-test); # - p = 0.07–0.09.

**Figure 2:**
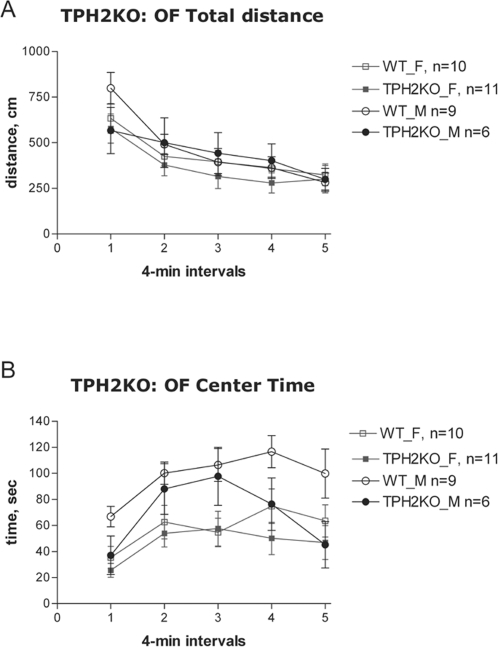
Open Field total distance (A) and center time (B) in TPH2KO mice. M – males, F- females.

Results from testing TPH2KO mice in the OF revealed that total distance traveled did not differ between KO and WT mice (Fig. 2A). There was a significant effect of time [F(4,128) = 50.5, P<0.00001] indicating habituation to the OF, evident in both genotypes. There was also a sex×genotype×time interaction [F(4,128) = 2.7, P<0.05] on total distance traveled; however, no sex differences were observed. There was a non-significant trend in both genders for the TPH2KO to spend less time in the center [F(1, 32) = 3.35, p = 0.076] and genotype×time interaction was significant [Fig. 2B, F(4,128) = 2.8, P<0.05]. There were significant sex effects on center time and center distance in both genotypes [F(1,32)>7.5, P<0.01] with males spending more time and traveling more distance in the center than females. There was no difference between TPH2KO and WT mice in exploratory behaviors such as hole-poke and rearing.

Similar to the results for the TPH2KO, there were no significant differences between DKO and WT mice in total distance traveled, indicating normal locomotor activity (Table 4). None of the effects or interactions approached significance in this data analysis. This is also consistent with the lack of differences between genotypes in the circadian activity assay (Table 4). There was no difference between genotypes in center time and exploratory behaviors between DKO and WT mice.

**Figure 3:**
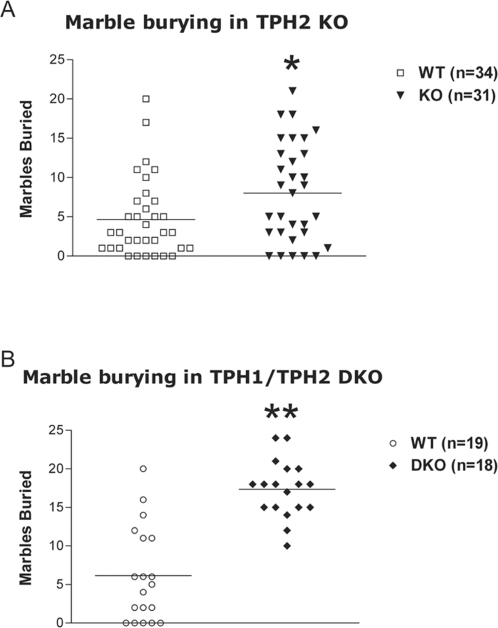
Marble burying test. Male and female data combined on the plots since one-way ANOVA did not reveal significant effect of sex or sex×genotype interactions. (A) Marble burying in TPH2KO mice. Males: 15 WT, 16 KO; females: 19 WT, 15 KO. (B) Marble burying in TPH1/TPH2 DKO mice. Males: 10 WT, 6 DKO; females: 9 WT, 12 DKO. * P<0.05 compared to WT mice, ** - p<0.0001 compared to WT mice.

The marble burying (novelty-induced digging) test is a valuable screening test for the detection of compounds having anxiolytic and/or antidepressant effects and is effective in detecting the behavioral effects of serotonin uptake inhibitors [29], [30]. In TPH2KO cohort 2, male KO mice buried significantly more marbles (P<0.05, data not shown). When marble burying was repeated in TPH2KO cohort 3 homozygous mice again buried significantly more marbles than WT mice (Fig. 3A, F(1, 61) = 5.61, P<0.05). There was no effect of sex and no genotype×sex interaction for marble burying in TPH2KO mice. There was a significant effect of genotype with DKO mice burying significantly more marbles than WT mice (Fig. 3B, F(1, 33) = 39.17, P<0.0001). There was no effect of sex and no genotype×sex interaction for marble burying in DKO mice.

**Figure 4:**
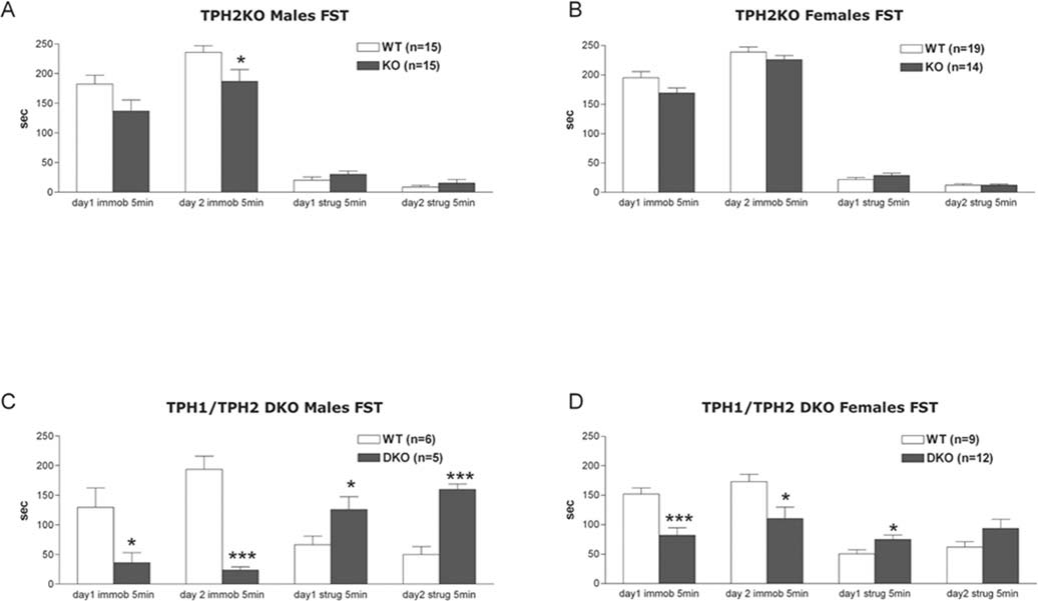
Immobility and struggling in the forced swim test. (A) TPH2KO Males. (B) TPH2KO Females. (C) TPH1/TPH2 DKO Males. (D) TPH1/TPH2 DKO Females. *** - p<0.001, ** - P<0.01, * - p<0.05 compared to WT mice (unpaired t-test).

In the forced swim test, male TPH2KO mice exhibited trends for decreased immobility on pre-test day (t(28) = 1.9, P = 0.068) and significantly less immobility on the test day (t(28) = 2.1, P<0.05, Fig. 4A). In female TPH2KO mice only a trend in the same direction was observed (Fig. 4B). There was no difference in struggling behavior between TPH2KO and WT mice. DKO males displayed less immobility on both pre-test (t(9) = 2.4, P<0.05) and test days (t(9) = 6.7, P<0.0001), and also more struggling on both pre-test (t(9) = 2.4, P<0.05) and test days (t(9) = 6.4, P<0.001, Fig. 4C). DKO females also showed significantly lower immobility on both pre-test (t(19) = 3.96, P<0.001) and test days (t(19) = 2.5, P<0.05, Fig. 4D). Similar to the males, DKO females struggled more than WT females on both days, but the difference reached significance only on pre-test day 1 (t(19) = 2.2, P<0.05). Overall, genetic disruption of TPH2 resulted in a mild antidepressant phenotype, and this became much more significant in the DKO mice. Visual observation of swimming behavior suggests that DKO mice had difficulty keeping their heads above water when not actively swimming. Video S1 showcases this inability to float in DKO mice (from left to right: WT, DKO, DKO).

**Figure 5:**
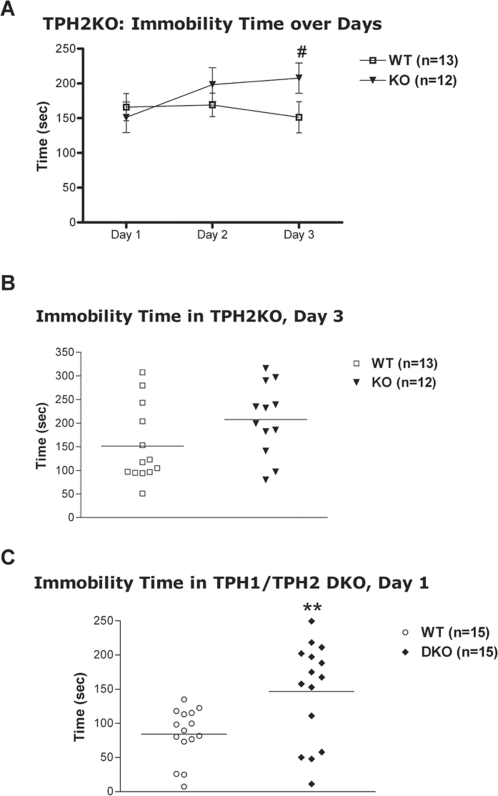
Tail suspension test. (A) Immobility time in TPH2KO male mice over days. # - p<0.05, compared to Day 1 for the same genotype (Tukey's HSD following repeated measures ANOVA). (B) Immobility time in TPH2KO male mice on day 3 of repeated TS testing. (C) Immobility time in TPH1/TPH2 DKO. Male and female data combined on the plot (one-way ANOVA did not reveal significant effect of sex or sex×genotype interactions). Males: 7 WT, 6 DKO; females: 8 WT, 9 DKO. Mice that climbed tails during testing were excluded from analysis. ** - p<0.01 compared to WT mice.

There was no significant effect of genotype in a standard one-day tail suspension testing in either male or female TPH2KO mice. Male mice from cohort 2 were additionally subjected to three days of repeated tail suspension testing. The results showed only a significant genotype×day interaction for immobility time [F(2,46) = 4.1, p<0.05), but still no genotype effect (Fig. 5A). Analysis of interaction showed that immobility significantly increased from day 1 to day 3 in TPH2KO (p<0.05 – immobility on day 3, compared to day 1) but did not change in the WT. TPH2KO tended to have higher immobility on day 3 than WT mice (Fig. 5B). In DKO mice there was a significant effect of genotype [F(1,26) = 8.0, p<0.01] and sex [F(1,26) = 6.0, p<0.05], but no genotype×sex interaction. DKO mice had significantly greater immobility time, compared to WT littermates (Fig. 5C). Female mice of both genotypes had higher immobility time than males.

**Figure 6:**
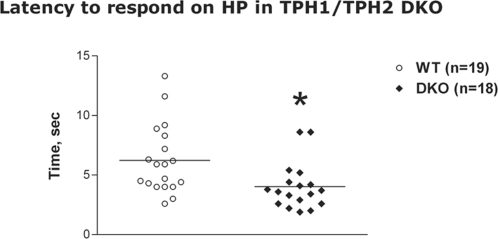
Latency to hind paw response in the hot plate acute thermal pain test in TPH1/TPH2 DKO mice. Male and female data combined on the plot (one-way ANOVA did not reveal significant effect of sex or sex×genotype interactions). Males: 10 WT, 6 DKO; females: 9 WT, 12 DKO. * - p<0.05, compared to WT mice.

Serotonergic systems are involved in the central regulation of nociceptive sensitivity. In our experiments there was no difference between TPH2 and DKO mutants and WT cohort-mates in tonic pain sensitivity assessed by formalin paw test (Table 4). There was no significant difference between genotypes for TPH2 KO mice in hot plate model. However, DKO mice exhibited altered pain sensitivity to an acute thermal stimulus. There was a significant effect of genotype with DKO having a lower latency to respond (Fig. 6, F(1,33) = 5.68, p<0.05). There was no effect of sex and no genotype×sex interaction for latency to respond on the hot plate in DKO mice. This data indicates that DKO mice had increased sensitivity to acute nociception in the hot plate assay.

## Discussion

When TPH1 was first genetically deleted in mice, it was generally expected this would result in homozygous lethality [20]. Surprisingly, those TPH1 homozygote mice appeared largely normal in appearance and behavior. Our results escalate this surprise further as genetic deletions of the neuronal enzyme TPH2 and the combination of TPH1 plus TPH2 enzymes also produced viable mice with relatively subtle alterations in behavior. The results dramatically indicate that 5-HT is not essential for overall development and that its role in behavior is modulatory rather than essential.

Initial phenotypic analysis of these mutants revealed no differences in a range of measures of physical health including assays for cardiac, immune system, endocrine, and ophthalmic function (unpublished observations). TPH2KO and DKO mice also did not differ from their WT cohort-mates in gross appearance, however TPH2KO and DKO males were smaller in body length and weight, and DKO males and females had significantly lower fat content, compared to WT controls. Recently Gutknecht *et. al.* [31] have reported similar general phenotypic observations of their TPH2 KO animals with detailed histological analysis showing no loss of serotonergic cells in the raphe nucleus of the brain in spite of total loss of 5-HT. Our data on the 5-HT level from TPH1KO mice [27] are consistent with results from a previous report [18], with blood and intestinal 5-HT levels greatly reduced, yet no alteration in 5-HT in the brain. TPH2KO mice did not differ significantly from WT mice in peripheral 5-HT levels, however levels of 5-HT and its metabolite, 5-HIAA, were strongly reduced in all brain regions examined. In DKO mice 5-HT levels were dramatically reduced in both peripheral and brain tissues. These data confirm the widely held hypothesis that TPH1 and TPH2 are the primary enzymes for 5-HT synthesis in peripheral tissues and in the central nervous system, respectively.

TPH2KO and DKO animals appear to contain residual 5-HT in the brain. In the TPH2KO mice this may represent inadvertent contamination from blood. However, small amounts were also seen in the DKO animals where peripheral 5-HT levels were negligible. It is possible this apparent 5-HT is due to another neurochemical with HPLC mobility very similar to 5-HT or to tryptophan hydroxylase-like activity by another enzyme such as phenylalanine hydroxylase [32].

Genetic deletion of 5-HT synthesis did not affect locomotion or exploratory behavior as no differences were found in the open field test, inverted screen, and circadian activity of all three mutants tested and compared to their WT cohort-mates. The trend for the reduction in center time in the TPH2KO may be an indicator of increased anxiety. Whole brain 5-HT levels are also reduced, almost 70%, in serotonin transporter (SERT) KO mice. Reduced activity and increased anxiety-like behavior was observed in the SERT KO mice in the novel open field, elevated plus-maze and light/dark exploration test [33], [34]. This is somewhat consistent with the findings in the present study although the SERT KO phenotype was more robust.

Compounds having anxiolytic or antidepressant effects alter behavior in MB, reducing the number of marbles that become buried [30], [35]. Acute treatment with pharmacologic inhibitors of SERT, selective serotonin reuptake inhibitors (SSRI) and less specific uptake inhibitors, results in a decreased number of marbles buried and is presumed to be indicative of their antidepressant or anxiolytic activity [29], [36]. Genetic deletion of SERT results in mice that bury fewer marbles [34]. The interpretation of increased marble burying found in the present study is not clear, but has been suggested to be a measure of obsessive/compulsive behavior rather than anxiety-like behavior [37]. It is possible that abnormality of this form of behavior, rather than altered anxiety, accounts for the increased marble burying in TPH2 and TPH1/TPH2 DKO mice.

Considering the established involvement of serotonergic mechanisms in the pathophysiology of depression, we evaluated the depressive-like behavior of mice in two common animal models used for antidepressant screening: the forced swim test (FST) and tail suspension (TS). The direction of effects in FST and TS were opposite in the KO animals, FST suggesting an antidepressant-like effect and TS exhibiting what might be a prodepressive-like effect. Visual observation suggests that DKO mice had difficulty keeping their heads above water when not swimming. This inability to float may contribute to the apparent antidepressant phenotype in the FST and explain the opposite direction of effects in the two tests. It is interesting to speculate whether the reduced body fat, therefore increased body density, in the DKO contributes to this difference in floating behavior.

In addition, although both TS and FST tests hold high predictive validity for potential antidepressants, they are not synonymous because distinct neurochemical and neuroanatomical pathways are involved [38], [39], [40]. Of particular relevance, FST produces a variation of the monoamine concentration in the brain while TS does not [40], [41]. In addition, serotonin and dopamine concentrations increased after FST, but no changes in neurotransmitter concentrations were observed after TS [40]. There are also inter-strain differences in mouse performance in these tests. For example, in NIH-Swiss mice, a seven-fold difference in baseline immobility was observed between the FST and TS. By contrast, the baseline immobility in C57Bl/6 mice was similar in both procedures. Also the antidepressant-like activity of various classes of antidepressants was only detected using the NIH-Swiss mice [39]. Higher levels of immobility in the TS test were also reported for serotonin transporter (SERT) KO mice on a 129 or C57BL/6Jx129S6 mixed genetic background [34], [42]. However, SERT KO mice backcrossed onto a C57BL/6J background were normal in acute testing in TS and FST [33]. In a recent report, a TPH2 knockin mouse line with reduced TPH2 activity and an 80% reduction in brain 5-HT, due to a rare human SNP (R441H), was reported to have significantly increased immobility times in the TS [43].

Serotonergic systems are involved in the central regulation of nociceptive sensitivity. Whether 5-HT has an analgesic or hyperalgesic action depends on the cell type and type of receptor it is acting through. In the periphery, 5-HT sensitizes afferent nerve fibers, thus contributing to hyperalgesia in inflammation and nerve injury [44]. Antidepressant drugs are reported to be used as co-analgesics in clinical management of migraine and neuropathic pain [45], [46]. Several studies have reported that a selective serotonin reuptake inhibitor, fluoxetine, produces an antinociceptive effect in pain assays, including the hot plate test [47], [48]. In our experiments, DKO animals exhibited altered pain sensitivity to an acute thermal stimulus in the hot plate model supporting 5-HT involvement in the regulation of pain.

In summary, the present study shows that genetic deletion of TPH2 strongly reduces the amount of 5-HT and 5-HIAA in the brain, but does not reduce their levels in the periphery. This is consistent with evidence that TPH2 is the primary synthetic enzyme in the brain, while TPH1 is responsible for 5-HT synthesis in extraneuronal tissues. The combined deletion of TPH1 and TPH2 resulted in a near total loss of 5-HT and its metabolite, 5-HIAA, in both the brain and periphery. The further reduction in brain 5HT in the DKO may suggest that TPH1 can provide some 5-HT in the absence of TPH2, either in situ or transposed from the blood.

5-HT is detected early during brain development, suggesting its involvement in neuronal proliferation, migration, and differentiation [49]. However, we detected no structural changes in the brain or peripheral tissues. It is notable that multiple 5-HT receptor null mutations that have been described, e.g. 5-HT1AR, 5-HT1BR, 5- HT2CR, 5-HT5AR, 5-HT2AR, 5- HT3R, and 5-HT6R, produce healthy animals with no overt abnormalities in appearance or behavior [1]. Similarly, genetic disruption of SERT also does not produce overt abnormalities [33], [34].

TPH2KO and DKO mice were generally normal in appearance and in many of the behavioral tests employed in this study. This stands in strong contrast to genetic deletion of tyrosine hydroxylase, the rate-limiting enzyme for catecholamine synthesis, which results in perinatal lethality [50], [51]. However, differences in behavior were evident in tests sensitive to serotonergic drugs like SSRIs and some compounds acting at 5-HT receptors. Interestingly, deletion of *Tph2* alone produced rather subtle behavioral changes; these were strongly enhanced in the DKO mice. Whether this difference was a result of *Tph1* providing supplementary 5-HT in brain regions where *Tph2* is predominant, or whether 5-HT normally synthesized in the brain by *Tph1* plays some particularly important role in control of behavior will require additional studies. Despite its peripheral expression, a polymorphism located on TPH1 has been reported to influence the response to antidepressant treatment [52]. Our results strongly support targeting the 5-HT system to treat affective disorders and the use of knockout mice as a tool to tease apart mechanisms involved in the etiology of these disorders. The availability of mice with genetic disruption of TPH2 and TPH1/TPH2 provides biologists with a set of important tools for better understanding the roles of serotonin in mammalian physiology and behavior.

## Supporting Information

Methods S1 Supplemental methods
(0.04 MB DOC)

Table S1 TPH2KO and TPH1/TPH2 DKO mice exhibit no changes in a wide number of tissues and organs examined - histological sections and necropsy
(0.03 MB DOC)

Table S2 An extensive battery used in standardized phenotypic evaluation.
(0.07 MB DOC)

Video S1 A representative example of day 2 FST performance. Male mice from left to right: WT, DKO, DKO
(4.75 MB MOV)

## References

[1] Bonasera SJ, Tecott LH (2000). Mouse models of serotonin receptor function: toward a genetic dissection of serotonin systems.. Pharmacol Ther.

[2] Buhot MC (1997). Serotonin receptors in cognitive behaviors.. Curr Opin Neurobiol.

[3] Ericsson M, Poston WS, Foreyt JP (1996). Common biological pathways in eating disorders and obesity.. Addict Behav.

[4] Goodman WK (1999). Obsessive-compulsive disorder: diagnosis and treatment.. J Clin Psychiatry.

[5] Julius D (1998). Serotonin receptor knockouts: a moody subject.. Proc Natl Acad Sci U S A.

[6] Mann JJ (1999). Role of the serotonergic system in the pathogenesis of major depression and suicidal behavior.. Neuropsychopharmacology.

[7] Gershon MD (2003). Serotonin and its implication for the management of irritable bowel syndrome.. Rev Gastroenterol Disord.

[8] Cote F, Fligny C, Bayard E, Launay JM, Gershon MD (2007). Maternal serotonin is crucial for murine embryonic development.. Proc Natl Acad Sci U S A.

[9] Coon H, Dunn D, Lainhart J, Miller J, Hamil C (2005). Possible association between autism and variants in the brain-expressed tryptophan hydroxylase gene (TPH2).. Am J Med Genet B Neuropsychiatr Genet.

[10] Sheehan K, Lowe N, Kirley A, Mullins C, Fitzgerald M (2005). Tryptophan hydroxylase 2 (TPH2) gene variants associated with ADHD.. Mol Psychiatry.

[11] Delorme R, Durand CM, Betancur C, Wagner M, Ruhrmann S (2006). No human tryptophan hydroxylase-2 gene R441H mutation in a large cohort of psychiatric patients and control subjects.. Biol Psychiatry.

[12] Haavik J, Blau N, Thony B (2008). Mutations in human monoamine-related neurotransmitter pathway genes.. Hum Mutat.

[13] McKinney J, Johansson S, Halmoy A, Dramsdahl M, Winge I (2008). A loss-of-function mutation in tryptophan hydroxylase 2 segregating with attention-deficit/hyperactivity disorder.. Mol Psychiatry.

[14] Sheehan K, Hawi Z, Gill M, Kent L (2007). No association between TPH2 gene polymorphisms and ADHD in a UK sample.. Neurosci Lett.

[15] Ballenger JC (1999). Current treatments of the anxiety disorders in adults.. Biol Psychiatry.

[16] Dodick DW, Sandrini G, Williams P (2007). Use of the sustained pain-free plus no adverse events endpoint in clinical trials of triptans in acute migraine.. CNS Drugs.

[17] Kugaya A, Seneca NM, Snyder PJ, Williams SA, Malison RT (2003). Changes in human in vivo serotonin and dopamine transporter availabilities during chronic antidepressant administration.. Neuropsychopharmacology.

[18] Walther DJ, Peter JU, Bashammakh S, Hortnagl H, Voits M (2003). Synthesis of serotonin by a second tryptophan hydroxylase isoform.. Science.

[19] Cote F, Thevenot E, Fligny C, Fromes Y, Darmon M (2003). Disruption of the nonneuronal tph1 gene demonstrates the importance of peripheral serotonin in cardiac function.. Proc Natl Acad Sci U S A.

[20] Walther DJ, Bader M (2003). A unique central tryptophan hydroxylase isoform.. Biochem Pharmacol.

[21] Wattler S, Kelly M, Nehls M (1999). Construction of gene targeting vectors from lambda KOS genomic libraries.. Biotechniques.

[22] Sambrook J, Fritsch E, Maniatis T (1989). Molecular Cloning: A Laboratory Manual..

[23] Martin JR, Bos M, Jenck F, Moreau J, Mutel V (1998). 5-HT2C receptor agonists: pharmacological characteristics and therapeutic potential.. J Pharmacol Exp Ther.

[24] Cryan JF, Valentino RJ, Lucki I (2005). Assessing substrates underlying the behavioral effects of antidepressants using the modified rat forced swimming test.. Neurosci Biobehav Rev.

[25] Drugan RC, Skolnick P, Paul SM, Crawley JN (1989). A pretest procedure reliably predicts performance in two animal models of inescapable stress.. Pharmacol Biochem Behav.

[26] Pogorelov VM, Baker KB, Malbari MM, Lanthorn TH, Savelieva KV, Kalueff A, LaPorte J (2008). A Standardized Behavioral Test Battery to Identify and Validate Targets for Neuropsychiatric Diseases and Pain.. Experimental models in neurobehavioral research.

[27] Liu Q, Yang Q, Sun W, Vogel P, Heydorn W (2008). Discovery and characterization of novel tryptophan hydroxylase inhibitors that selectively inhibit serotonin synthesis in the gastrointestinal tract.. J Pharmacol Exp Ther.

[28] Veenstra-VanderWeele J, Cook EH (2003). Knockout mouse points to second form of tryptophan hydroxylase.. Mol Interv.

[29] Li X, Morrow D, Witkin JM (2006). Decreases in nestlet shredding of mice by serotonin uptake inhibitors: comparison with marble burying.. Life Sci.

[30] Nicolas LB, Kolb Y, Prinssen EP (2006). A combined marble burying-locomotor activity test in mice: a practical screening test with sensitivity to different classes of anxiolytics and antidepressants.. Eur J Pharmacol.

[31] Gutknecht L, Waider J, Kraft S, Kriegebaum C, Holtmann B (2008). Deficiency of brain 5-HT synthesis but serotonergic neuron formation in Tph2 knockout mice.. J Neural Transm.

[32] Renson J, Weissbach H, Udenfriend S (1962). Hydroxylation of tryptophan by phenylalanine hydroxylase.. J Biol Chem.

[33] Holmes A, Yang RJ, Murphy DL, Crawley JN (2002). Evaluation of antidepressant-related behavioral responses in mice lacking the serotonin transporter.. Neuropsychopharmacology.

[34] Zhao S, Edwards J, Carroll J, Wiedholz L, Millstein RA (2006). Insertion mutation at the C-terminus of the serotonin transporter disrupts brain serotonin function and emotion-related behaviors in mice.. Neuroscience.

[35] Millan MJ, Brocco M, Papp M, Serres F, La Rochelle CD (2004). S32504, a novel naphtoxazine agonist at dopamine D3/D2 receptors: III. Actions in models of potential antidepressive and anxiolytic activity in comparison with ropinirole.. J Pharmacol Exp Ther.

[36] Kobayashi T, Hayashi E, Shimamura M, Kinoshita M, Murphy NP (2008). Neurochemical responses to antidepressants in the prefrontal cortex of mice and their efficacy in preclinical models of anxiety-like and depression-like behavior: a comparative and correlational study.. Psychopharmacology (Berl).

[37] Londei T, Valentini AM, Leone VG (1998). Investigative burying by laboratory mice may involve non-functional, compulsive, behaviour.. Behav Brain Res.

[38] Bai F, Li X, Clay M, Lindstrom T, Skolnick P (2001). Intra- and interstrain differences in models of “behavioral despair”.. Pharmacol Biochem Behav.

[39] Bourin M, Chenu F, Ripoll N, David DJ (2005). A proposal of decision tree to screen putative antidepressants using forced swim and tail suspension tests.. Behav Brain Res.

[40] Renard CE, Dailly E, David DJ, Hascoet M, Bourin M (2003). Monoamine metabolism changes following the mouse forced swimming test but not the tail suspension test.. Fundam Clin Pharmacol.

[41] Yadid G, Overstreet DH, Zangen A (2001). Limbic dopaminergic adaptation to a stressful stimulus in a rat model of depression.. Brain Res.

[42] Lira A, Zhou M, Castanon N, Ansorge MS, Gordon JA (2003). Altered depression-related behaviors and functional changes in the dorsal raphe nucleus of serotonin transporter-deficient mice.. Biol Psychiatry.

[43] Beaulieu JM, Zhang X, Rodriguiz RM, Sotnikova TD, Cools MJ (2008). Role of GSK3 beta in behavioral abnormalities induced by serotonin deficiency.. Proc Natl Acad Sci U S A.

[44] Sommer C (2006). Is serotonin hyperalgesic or analgesic?. Curr Pain Headache Rep.

[45] Wallace JM (2007). Update on pharmacotherapy guidelines for treatment of neuropathic pain.. Curr Pain Headache Rep.

[46] Leo RJ, Brooks VL (2006). Clinical potential of milnacipran, a serotonin and norepinephrine reuptake inhibitor, in pain.. Curr Opin Investig Drugs.

[47] Begovic A, Zulic I, Becic F (2004). Testing of analgesic effect of fluoxetine.. Bosn J Basic Med Sci.

[48] Singh VP, Jain NK, Kulkarni SK (2001). On the antinociceptive effect of fluoxetine, a selective serotonin reuptake inhibitor.. Brain Res.

[49] Lauder JM (1993). Neurotransmitters as growth regulatory signals: role of receptors and second messengers.. Trends Neurosci.

[50] Kobayashi K, Morita S, Sawada H, Mizuguchi T, Yamada K (1995). Targeted disruption of the tyrosine hydroxylase locus results in severe catecholamine depletion and perinatal lethality in mice.. J Biol Chem.

[51] Zhou QY, Quaife CJ, Palmiter RD (1995). Targeted disruption of the tyrosine hydroxylase gene reveals that catecholamines are required for mouse fetal development.. Nature.

[52] Serretti A, Benedetti F, Zanardi R, Smeraldi E (2005). The influence of Serotonin Transporter Promoter Polymorphism (SERTPR) and other polymorphisms of the serotonin pathway on the efficacy of antidepressant treatments.. Prog Neuropsychopharmacol Biol Psychiatry.

