# Supplementary material for: Genetic Disruption of Both Tryptophan Hydroxylase Genes Dramatically Reduces Serotonin and Affects Behavior in Models Sensitive to Antidepressants
Source: PLoS One. 2008 Oct 15;3(10):e3301. doi: 10.1371/journal.pone.0003301 (PMC2565062; doi:10.1371/journal.pone.0003301)
Supplement: Methods S1 — Supplemental methods (0.04 MB DOC) [file pone.0003301.s001.doc]

**Methods S1**

**Behavior assays**

**Preliminary behavioral screen.** All mice were assessed for physical health, sensory, motor, and neurological function using a subset of tests derived from the Irwin screen (Irwin, 1968). General physical health and empty cage behaviors were observed by placing the mouse in an empty home cage for 60 sec. Afterwards, the mouse was lifted by the tail and observed for limb splaying and forepaw and hindpaw clutching for 20 sec.

**Circadian Activity.** The light/dark cycles were set to 7 a.m./7 p.m. Female mice were placed in individual cages at 4 p.m. and tested for seven consecutive days. Fine, ambulatory, and total activity was measured by a San Diego Instruments cage rack Photobeam Activity System (PAS-Home Cage, San Diego Instruments, San Diego, CA). Upon completion of testing, mice were returned to their original home-cage environments to acclimate to group housing for 7 days prior to starting additional behavioral analysis.

**Stress-Induced Hyperthermia (SIH) and Basal Body Temperature.** Body temperature measurements were recorded with a Physitemp Instruments TH-5 Thermalert and RET-3 rectal probes. Male wildtype and homozygous mice were tested in their colony room to prevent additional stress from transporting cages. Basal body temperatures were recorded as T_1_. Ten minutes later the stress-enhanced body temperature was recorded and designated T_2_. The difference between T_2_ and T_1_ is calculated as delta T (T_2_-T_1_ = ΔT). Only basal body temperatures were recorded for females.

**Inverted Screen.** For motor/strength coordination mice were tested with the inverted screen. Untrained mice were placed individually on top of a square wire screen (7.5 cm x 7.5 cm) mounted horizontally on a metal rod. The rod was then rotated 180 degrees so that the mice were on the bottom of the screens. The following behavioral responses were recorded over a 1-minute testing session: fell off, did not climb, and climbed up. The results were expressed as a ratio.

**Acoustic startle and prepulse inhibition of the startle response.** The apparatus consisted of twelve acoustic startle chambers for mice (SR-LAB, San Diego Instruments, San Diego, CA). The SR-LAB cylindrical animal enclosure monitors animal movements with an ultra-stable, hermetically sealed motion sensor using full 12 bit resolution (a range from zero to over 5000 millivolts) for accurately measuring the wide range of startle responses. Animals were individually placed into enclosures inside the sound-attenuated startle chambers and acclimated for 3 minutes. PPI was assessed in a test session lasting for 15 min, in which the subjects were presented with a series of discrete trials comprising a mixture of six types of trials: startle pulse (120 dB, for 40 ms), no stimulus (70 dB background noise), or prepulse stimulus of one of four sound levels (74, 78, 82 or 90 dB) for 20 ms, followed 100 ms later by an acoustic startle (120 dB for 40 ms). A total of 6 trials under each condition were delivered in a random sequence and all trials were separated by a variable inter-trial interval of 10–30 s. Max response to the stimulus (V max) was averaged for each trial type. In the startle alone trials, the basic auditory startle (startle response) was measured. Animals with an average startle response value equal to or below 100 were excluded from PPI analysis. In “no stimulus” trials a baseline measure was taken to assess movement in the enclosure under no stimulation. In the prepulse plus startle trials, PPI was calculated as a percentage score for each acoustic prepulse trial type using the formula: % PPI = 100−[((startle response for prepulse + pulse)/(startle response for pulse alone))] x100.

**Trace Fear conditioning.** Trace fear conditioning was carried out using eight conditioning chambers (Coulbourn Instruments, Allentown, USA). On the training day mice were placed in the conditioning chamber, and left to acclimate to the testing environment for 60 seconds. Then a conditioned stimulus (CS: 15 sec duration, 85 dB 3kHz) generated by a tone was delivered, followed by a trace period of 10 sec and the unconditioned stimulus (US: foot shock, 2 sec, 0.36 mA). Mice were presented with 5 trials with inter-trial interval (ITI) 180 seconds, and returned to the home cage 1 minute after the final shock. Memory for context-shock association was assessed 24 hours later. Each mouse was placed back into the same conditioning chamber for 5 minutes, and freezing was recorded using a video camera and Actimetrics FreezeFrame software (Actimetrics, Inc., Wilmette, IL). Memory for tone-shock association was assessed on the following day (48 hours after training) in a different room and chamber environment, in order to assess the efficacy of cue (tone) conditioning in the absence of interference from context conditioning. Each mouse was placed into a chamber that differed from conditioning chambers in lighting conditions (red instead of white), walls (black instead of clear) and flooring (rough plexi-glass instead of metal grid). After 60 seconds (Pre-CS), the CS (same tone as in training) was presented for 15 seconds, followed by 60 seconds without tone (Post-CS). Memory for cued fear conditioning was assessed as a difference in percentage of time that mice spent freezing after the tone (Post-CS, conditioned freezing) and the percentage of time they spent freezing before the tone (Pre-CS, baseline freezing).

**Formalin Paw.** Mice were tested for nociception with Automatic Nociception Analyzers (purchased from the Ozaki lab at University of California, San Diego). A metal band was placed around the left hind paw of each mouse with superglue 30 minutes prior to testing. After the 30-minute acclimation period, 20 µl of 5% formalin was subcutaneously injected in the dorsal surface of the left hind paw. Mice were individually housed in cylindrical chambers for 45 minutes. A computer recorded flinches per minute, total flinches for Phase I (acute phase = first 8 minutes), and total flinches for Phase II (tonic phase between 20 - 40 minutes) through an electromagnetic field.
