# Supplementary material for: Genetic Disruption of Both Tryptophan Hydroxylase Genes Dramatically Reduces Serotonin and Affects Behavior in Models Sensitive to Antidepressants
Source: PLoS One. 2008 Oct 15;3(10):e3301. doi: 10.1371/journal.pone.0003301 (PMC2565062; doi:10.1371/journal.pone.0003301)
Supplement: Table S1 — TPH2KO and TPH1/TPH2 DKO mice exhibit no changes in a wide number of tissues and organs examined - histological sections and necropsy (0.03 MB DOC) [file pone.0003301.s002.doc]

**Table S1.** TPH2KO and TPH1/TPH2 DKO mice exhibit no changes in a wide number of tissues and organs examined - histological sections and necropsy

| Necropsy | Rectum | Salivary gland | Vas deferens |
| --- | --- | --- | --- |
| Heart | Mesenteric nodes | Pituitary | Prostrate gland |
| Aorta | Liver | Esophagus | Fallopian tubes |
| Skeletal muscle | Gallbladder | Stomach | Ovaries |
| Lung | Spleen | Pancreas | Testes with epididymis |
| Kidneys | Brain | Duodenum | Skin |
| Trachea | Eyes | Jejunun | Mammary gland |
| Thyroid | Harderian glands | Ileum | Inguinal node |
| Parathyroids | Urinary bladder | Neonate/whole body | Femur |
| Adrenal glands | Uterine | Gross | Sternum |
| Thymus | Seminal vesicles | Embryo | Blood cell compartments |
