# Supplementary material for: Genetic Disruption of Both Tryptophan Hydroxylase Genes Dramatically Reduces Serotonin and Affects Behavior in Models Sensitive to Antidepressants
Source: PLoS One. 2008 Oct 15;3(10):e3301. doi: 10.1371/journal.pone.0003301 (PMC2565062; doi:10.1371/journal.pone.0003301)
Supplement: Table S2 — An extensive battery used in standardized phenotypic evaluation. (0.07 MB DOC) [file pone.0003301.s003.doc]

**Table S2.** An extensive battery used in standardized phenotypic evaluation**.**
